# *Arabidopsis* Natural Accessions Display Adaptations in Inflorescence Growth and Vascular Anatomy to Withstand High Salinity during Reproductive Growth

**DOI:** 10.3390/plants8030061

**Published:** 2019-03-11

**Authors:** Sahar Sellami, Rozenn Le Hir, Michael R. Thorpe, Emilie Aubry, Nelly Wolff, Françoise Vilaine, Faiçal Brini, Sylvie Dinant

**Affiliations:** 1 Institut Jean-Pierre Bourgin, INRA, AgroParisTech, CNRS, Université Paris-Saclay, 78000 Versailles, France; sahar.sellami@inra.fr (S.S.); rozenn.le-hir@inra.fr (R.L.H.); Emilia.Aubry@inra.fr (E.A.); nellywolff8@gmail.com (N.W.); francoise.vilaine@inra.fr (F.V.); 2 Biotechnology and Plant Improvement Laboratory, Center of Biotechnology of Sfax, (CBS)/University of Sfax, 3018 Sfax, Tunisia; faical.brini@cbs.rnrt.tn; 3 Plant Science Division, Research School of Biology, The Australian National University, Canberra, ACT 0200, Australia; michael.thorpe@anu.edu.au

**Keywords:** salt stress, carbon allocation, inflorescence, vascular tissues, transport, phloem, xylem, hydraulic conductivity, irregular xylem vessels, irx

## Abstract

Plant responses to abiotic stresses entail adaptive processes that integrate both physiological and developmental cues. However, the adaptive traits that are involved in the responses to a high soil salinity during reproductive growth are still poorly studied. To identify new clues, we studied the halophyte, *Thellungiella salsuginea*, and three *Arabidopsis* accessions, known as tolerant or salt-sensitive. We focused on the quantitative traits associated with the stem growth, sugar content, and anatomy of the plants subjected to the salt treatment, with and without a three-day acclimation, applied during the reproductive stage. The stem growth of *Thellungiella salsuginea* was not affected by the salt stress. By contrast, salt affected all of the *Arabidopsis* accessions, with a natural variation in the effect of the salt on growth, sugar content, and stem anatomy. In response to the high salinity, irregular xylem vessels were observed, independently of the accession’s tolerance to salt treatment, while the diameter of the largest xylem vessels was reduced in the tolerant accessions. The stem height, growth rate, hexoses-to-sucrose ratio, and phloem-to-xylem ratio also varied, in association with both the genotype and its tolerance to salt stress. Our findings indicate that several quantitative traits for salt tolerance are associated with the control of inflorescence growth and the adjustment of the phloem-to-xylem ratio.

## 1. Introduction

Salinity is one of the major environmental abiotic constraints limiting crop productivity. The salinization of arable soils first occurs because of seawater incursions and sea winds. It can also result from the accumulation of soluble salts arising from high rates of evaporation, or from excessive irrigation when a high water table or slow drainage lead to the secondary salinization of arable lands. Deforestation for extensive cropping has also been shown to cause a rising of the saline water table. Depending on the weather and on agricultural practices, plants can therefore face a high salinity at different stages of their development. Responses in the seedling and vegetative stages have been described in detail for many plant species, including *Arabidopsis* [1,2], and several reports suggested that in their early vegetative stages, plants are particularly susceptible [1]. In contrast, the reproductive stage’s responses have not yet been evaluated as extensively. 

The response to high salinity follows two-phases, as follows: first, a rapid osmotic phase, followed by a secondary ionic phase due to the toxic effect of the accumulation of Na^+^ ions [2]. These combined effects can severely alter the growth of the glycophytic species, although less so in halophytic species. Plants employ many strategies to survive these two phases of salt stress, to maintain their homeostasis, and tolerate ion toxicity. These strategies rely on the major transcriptional reprogramming of the expression of the genes involved in the tolerance to osmotic stress, in the accumulation of Na^+^ ions, in the excessive accumulation of reactive oxygen species, and in the increased accumulation of compatible solutes [3,4,5,6]. Epigenetic chromatin modifications are also involved [7]. The uptake, translocation, storage, or exclusion of Na^+^ also changes [8]. There is a reduced translocation of Na^+^ ions towards the shoot via the xylem [2,9], and Na^+^ ions can be sequestered at a cell level in the roots and shoots by accumulation in the vacuole [8,10]. A general consequence of salt stress in the shoot is the decrease of the stomatal aperture, which in turn impairs carbon fixation and sugar allocation to the roots [2,9,11].

The accumulation of compatible solutes is a key feature for salt tolerance [2], with soluble sugars such as sucrose commonly accumulating [2,12]. Sugars can act as osmolytes to maintain cell turgor under increased water stress, and also lead to changes in the metabolism that protect the membranes and proteins from stress damage. These functions are intrinsically linked to the metabolic role of sugars for promoting faster growth to face stresses, by triggering developmental processes and fueling primary metabolism [13]. In glycophytic Arabidopsis, a natural variability has been shown during the vegetative stage in response to a high salinity, indicating that different adaptive processes are responsible for the tolerance [14,15,16,17,18]. Comparative metabolomic studies with the halophyte *Thellungiella salsuginea* (salt cress), a close relative of *Arabidopsis*, indicated a higher osmo-compatibility of *Thellungiella* metabolome [19], suggesting that this halophyte species is metabolically primed for osmoprotective strategies [12,20]. 

In taller plants, the stem acts as a buffer to provide water, ions, and nutrients when their uptake and utilization are not equal, as well as housing the xylem and phloem pathways for their longitudinal transport. Both transport processes can be altered by osmotic stresses, including drought and high salinity [11]. Recent studies further established that recovery after a prolonged drought depends on the stem concentration of the soluble carbohydrates [21,22]. It was also shown for *Arabidopsis* that the growth of the inflorescence can be affected by the temperature [23], as well as by a high salinity, associated with modifications of the lignin content and oxidative stress in the stem [24].

In *Arabidopsis*, major transitions occur during the development of the floral stem [25,26]. Although many studies of *Arabidopsis* physiology and nutrition have studied the vegetative stages, it is worth noting that the inflorescence stems can contribute more than the rosette to the lifetime carbon gain [27,28], and promote seed production directly [29]. Nevertheless, the growth of the inflorescence stem can be reduced and even stopped by the abiotic stresses that occur during its formation. For example, a drought treatment applied just after the onset of flowering can cause an early arrest of floral development, leading to sterility [30].

The aim of this study was to evaluate the response of several *Arabidopsis* accessions to high salinity during their reproductive stage, in order to explore the relationships between salt tolerance, sugar content, and the anatomy of the vascular tissues. *Arabidopsis* represents a glycophytic model species for exploring natural variation. For comparison, we included the halophyte species *Thellungiella salsuginea* in our analysis. The overall work provides clues on how stem growth and stem anatomy can contribute to the adaptation to salt stress.

## 2. Results

### 2.1. Experimental Scheme for Studying the Tolerance to High Salinity during the Reproductive Stage

**Figure 1:**
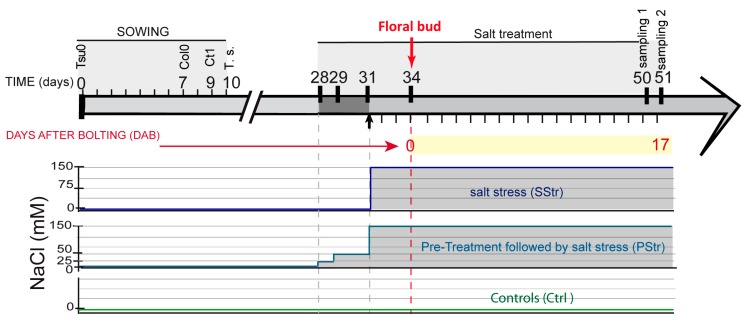
Design of the experimentation. Staggered sowing of the genotypes, with Tsushima-0 (Tsu0) sown at day 0, then Columbia-0 (Col0), Catania-1 (Ct1), and *Thellungiella salsuginea* (T.s.) sown 7, 9, and 10 days later, respectively, so as to synchronize bolting. Application of the salt treatment started three days before the floral bud appearance. The pretreatment phase (i.e., acclimation) of 150 mM of salt treatment preceded the beginning by three days. Sampling of the rosette and floral stem for the measurement of the fresh weight and dry weight was done at 16 days after bolting (DAB). Sampling of the main floral stem for anatomic studies and sugar quantifications was done at 17 DAB. Measurements of the stem height were done from 7 DAB to 16 DAB.

For the analysis of the tolerance to salt treatment during the reproductive stage, the halophyte *Thellungiella salsuginea* (salt cress) and three *Arabidopsis* accessions, Columbia-0 (Col0), Catania-1 (Ct1), and Tsushima-0 (Tsu0), were studied. The salt cress was chosen because of its well-known tolerance to abiotic stress, including salt treatment [20]. The *Arabidopsis* accessions Tsu0 and Ct1 were chosen based on their tolerance to high salinity at vegetative stages [15,16], while Col0 is considered as a salt sensitive accession [18]. The salt treatment was done by watering with 150 mM NaCl, which is sufficient to induce a stress response on the salt tolerant *Thellungiella* plants, without provoking the death of the *Arabidopsis* plants [19,31]. In order to allow the plants to form a floral stem under a high salinity, we designed a scheme that enabled the entire development of the floral stem under stress (Figure 1). Briefly, the plants were allowed to grow under normal conditions during rosette growth. Three days before bolting would occur, defined by the emergence of the floral bud, one third of the plants continued under normal conditions (control plants—Ctrl), whereas another third of the plants were watered with a solution containing 150 mM of NaCl (salt stress—SStr). The final third of the plants were submitted to the same salt treatment, but, in the preceding three days, they were subjected to a progressive increase in salt stress (pretreatment before stress—PStr).

### 2.2. Growth of *Thellungiella salsuginea* under High Salinity during the Reproductive Stage

**Figure 2:**
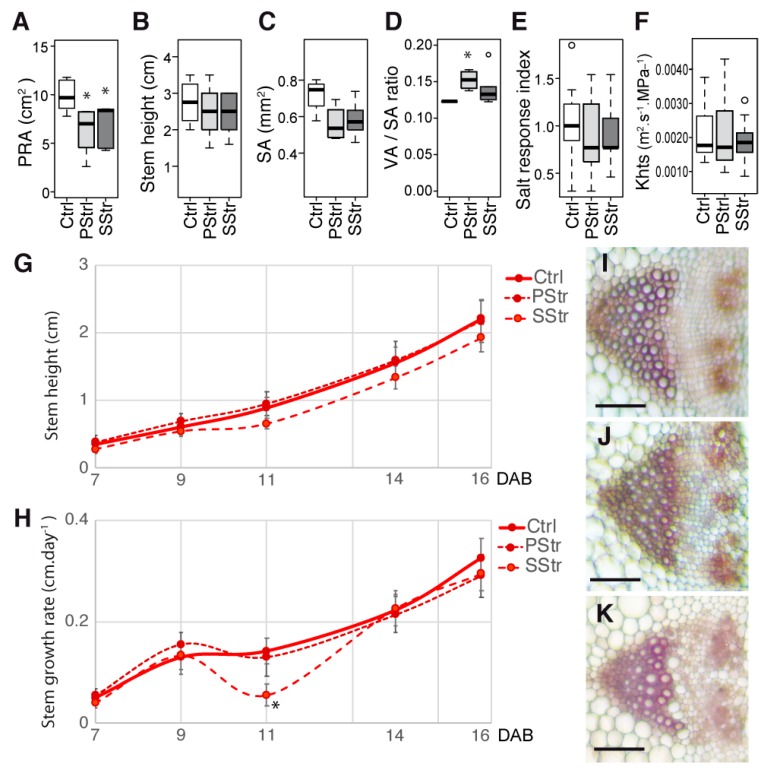
Responses of *Thellungiella salsuginea* to high salinity during the reproductive stage. Growth and anatomy of the stem of the control (Ctrl), salt stress (SStr), and pretreatment before stress (PStr) plants. In (**A**–**F**), the box and whisker plots show the distribution of the biological replicates. The black lines inside represent the medians; the top and bottom ends of the boxes represent the first and third quartiles, respectively; and the whisker extremities represent the maximum and minimum data points. (**A**) Projected rosette area at 17 DAB *(n* = 5–6). (**B**) Stem height at 17 DAB *(n* = 5–6). (**C**) Stem section area (SA) ratio at 17 DAB *(n* = 4–5). (**D**) Total vascular bundles area per section (VA)/stem section area (SA) ratio at 17 DAB *(n* = 4–5). (**E**) Salt response index at 16 DAB *(n* = 9–12). (**F**) Stem theoretical hydraulic specific conductivity (Khts) ratio at 17 DAB *(n* = 3–6). (**G**,**H**) Stem height and growth rate from 7 to 16 DAB. The curves represent for each time point the mean ± standard error (SE) (*n* = 9–12). (**I**,**J**,**K**) Details of the stem vascular bundles of the Ctrl plants (**I**), PStr plants (**J**), and SStr plants (**K**). Bar = 50 µm. Star (*) on the curves indicates a *p* < 0.05 with a t-test.

For the halophyte *Thellungiella salsuginea*, we observed an effect of the high salinity on the projected rosette area (Figure 2A), both in the PStr and SStr plants, but not on the stem length at 17 days after bolting (DAB) (Figure 2B), even though both the stem section area (Figure 2C) and the proportion of vascular tissue area per stem section area (Figure 2D) were affected in the salt-treated plants. It is worth noting that in these conditions, the stem growth was slow, its height reaching two to three cm at 16 DAB. A salt response index (SRI) of the stem was calculated, by dividing the mean stem growth rate of the plants grown under salt treatment by the growth rate in the normal conditions (equivalent to the osmotic tolerance index reported in the literature [32]). In the Ctrl and salt-treated plants, the stem growth was not affected under the salt treatment, which resulted in a salt response index close to 1 (Figure 2E). We also looked in the xylem for whether the salt treatment had an effect on the xylem vessels. We observed no differences in the dimensions of the vessels of the salt-treated plants, compared to the Ctrl plants, with a mean diameter of the largest vessels being approximately 8 µm (7.8 µm ± 1.6, *n* = 51), and, consequently, having no effect on the theoretical hydraulic specific conductivity that we deduced from the mean diameter (Figure 2F), with an average predicted value of 0.002 m^2^. s^−1^. MPa^_1^ (+/− 0.0007, *n* = 60). No differences were observed in the stem height from 7 to 16 DAB in the salt-treated plants compared to the Ctrl plants (Figure 2G). The growth rate of the stem remained unchanged, except at 11 DAB for the SStr plants, for which we observed a temporary, slight reduction (Figure 2H). Finally, the organization of the vascular bundles was similar in all three of the conditions (Figure 2I–K). 

### 2.3. Natural Variation in the Floral Stem Growth and in Sugar Content in Control Conditions

**Figure 3:**
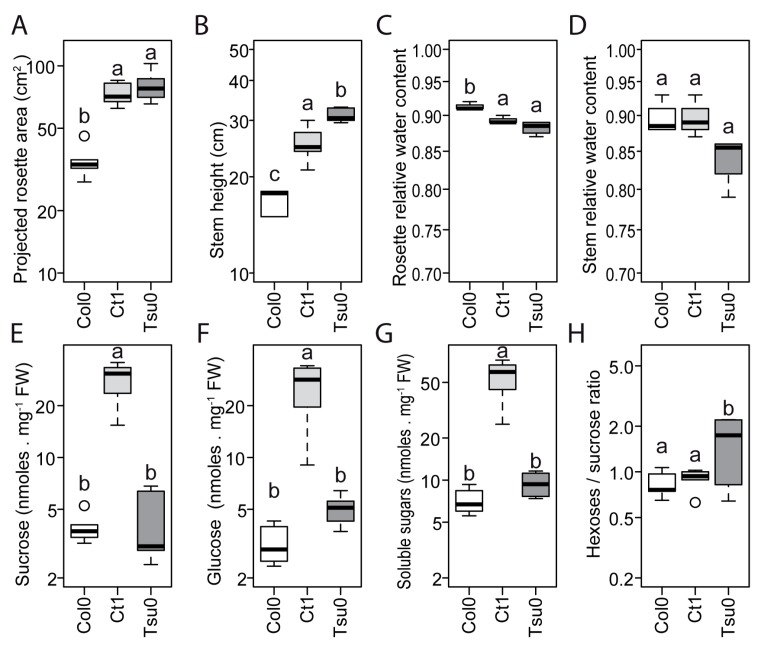
Natural variation in the size and sugar content of the Ctrl *Arabidopsis* plants. (**A**–**H**) The box and whisker plots represent the distribution of the biological replicates (see Figure 2 for details) with in (**A**) the projected rosette area (*n* = 5–6), (**B**) stem height (*n* > 9), (**C**) rosette relative water content (*n* = 4), (**D**) stem relative water content (*n* = 4), (**E**) sucrose content, (**F**) glucose content, (**G**) total soluble sugar content (i.e., sucrose, glucose, and fructose), and (**H**) and hexoses-to-sucrose ratio ((**E**–**H**): *n* = 5–6). All of the data were obtained at 17 DAB, except for the rosette and stem relative water content that were measured at 16 DAB. Different letters indicate significant differences among the accessions determined using a one-way analysis of variance (ANOVA), combined with a Tukey’s comparison post-test (*p* < 0.05).

In the control conditions, the projected rosette area and stem height of the *Arabidopsis* accessions differed (Figure 3A,B). The relative water content of the rosette, but not of the stem, was also significantly different between the different accessions (Figure 3C,D). The relative water content was significantly higher in the Col0 than in Ct1 and Tsu0 plants. The fructose content was low for all of the three genotypes (data not shown), while the sucrose and glucose contents differed between the accessions (Figure 3E,F,G). Th total soluble sugar content (fructose, glucose, and sucrose) was higher in the Ct1 plants than in the other accessions (Figure 3G). We also observed a variation in the hexoses-to-sucrose ratio (Figure 3H), with a hexoses-to-sucrose ratio higher in the Tsu0 plants. These data indicate diversity in the growth of the vegetative and reproductive organs, and in their sugar accumulation. Broad diversity in the sugar content of the rosette of the *Arabidopsis* accessions has been reported in a broad range of conditions [33,34], but so far, not for the stem. 

### 2.4. Diversity in the *Arabidopsis* Floral Stem Growth and Sugar Content in Response to High Salinity

**Figure 4:**
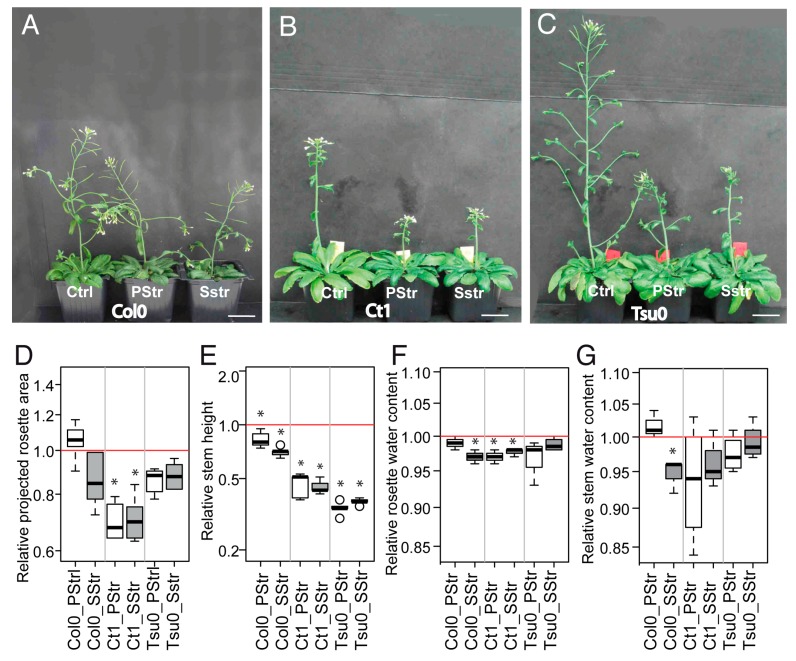
Physiological responses to salt treatment in *Arabidopsis* accessions. (**A**–**C**) Plant growth at 17 DAB under Ctrl, PStr, and SStr treatments, for the Col0 (**A**), Ct1 (**B**), and Tsu0 (**C**) plants. Scale bar = 2.5 cm. (**D**–**G**) The box and whisker plots represent the distribution of the biological replicates (see Figure 2 for details) within the (**D**) fold-changes in the projected rosette area at 17 DAB (*n* = 5–6), (**E**) the fold-changes in the stem height at 17 DAB (*n* > 6), (**F**) the fold-changes in rosette water content at 16 DAB (*n* = 4), and (**G**) the fold-changes in the stem water content at 16 DAB (*n* = 4). The fold changes were determined using the values that were normalized to the mean of control plants of the same accession. Red lines indicate the relative control means (= 1). Stars denote significant differences of treatments compared to the control plants (* *p* < 0.05, *n* ≥ 5). White bars—PStr plants. Grey bars—SStr plants.

When applying a salt stress during the reproductive stage, we observed an effect on the growth of both the rosette and the floral stem (Figure 4A–C), with a magnitude that depended on the accession. The projected rosette area (PRA) was smaller in the salt-treated Ct1 plants compared with the control Ct1 plants, in contrast to the rosette of Col0 and Tsu0 accessions, for which only a tendency for a smaller PRA was measured (Figure 4D). The stem was shorter in the salt-treated plants than in the Ctrl plants for all three accessions (Figure 4E). But the effects of the salt-treatment, with or without a pre-treatment, were much more pronounced at 16 DAB for the Ct1 and Tsu0 plants (50% and 65% shorter, respectively) than for the Col0 plants (30% shorter). We also observed some variations in the water content of the rosette and stem of the salt-treated plants compared with the Ctrl plants (Figure 4F,G). Regarding pre-treatment, comparing the SStr and PStr plants (Figure 4A–G), we observed no difference in either the growth or water content of any accession, except for the water content in the rosette and stem of the Col0 plants. Overall, these data indicate larger effects on the stem growth of the two tolerant accessions of Ct1 and Tsu0.

**Figure 5:**
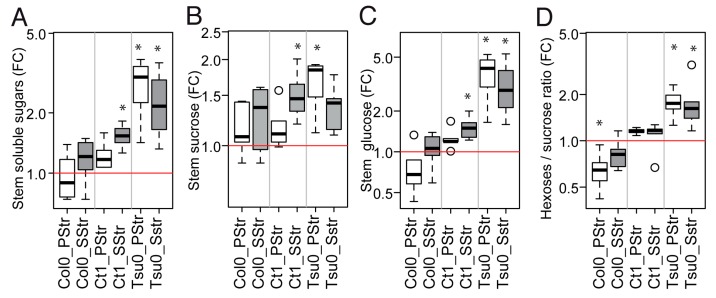
The natural variation of the response of the sugar content to salt treatment. Fold changes in the stem sugar contents in the PStr and SStr plants at 17 DAB. (**A**–**D**) The box and whisker plots represent the distribution of the biological replicates (see Figure 2 for details) with in (**A**) the fold changes in total soluble sugars, (**B**) the fold changes in sucrose, (**C**) the fold changes in glucose, and (**D**) the fold changes in hexoses-to-sucrose ratio (*n* = 5–6). FC—fold changes. Stars denote the significant differences of the treatments compared to the control plants (* *p* < 0.05, *n* ≥ 5). White bars—PStr plants. Grey bars—SStr plants.

Because the total soluble sugars content of the stem under normal conditions depended on accession (Figure 3E–H), we also analyzed the sugar content in the stem of the salt-treated plants (Figure 5). Significant fold-changes in the sugars content were observed in response to high salinity, depending on the accession (Figure 5A–D). Overall, the total soluble sugar content tended to be higher in the salt-treated plants than in the Ctrl plants, with the highest accumulation in Tsu0, with a five-fold change in the total soluble sugars and a three-fold change in the glucose content (Figure 5A,C). Interestingly, the hexoses-to-sucrose ratio was higher in the Tsu0 salt-treated plants than in the Ctrl plants. It was significantly lower in the PStr Col0 plants compared with the Ctrl plants. The ratio in the Ct1 salt-treated plants was similar to the one observed in the Ctrl plants (Figure 5D). Thus, the reduction in the stem growth under a high salinity was associated with variations in the sugar content, and contrasted values in the hexoses-to-sucrose ratio. This ratio has sometimes been considered as a proxy for a plant’s physiological and developmental status toward biomass production versus storage, with low ratios being associated with a high demand for cell division or expansion, and high ratios being associated with storage [35]. The differences in the accumulation of sugars in the stem could reflect the differences in its capacity to fix and/or export sugars at the stage of sampling.

Our data showed higher hexoses-to-sucrose ratios in the salt-treated Tsu0 plants, which could indicate a higher capacity to store soluble sugars at this developmental stage, while the lower ratio observed in the PStr Col0 plants could indicate higher demands for cell division or elongation. Whether this would also reflect the differences in the overall development of the inflorescence in the three accessions, and the faster or slower transitions from sink-to-source is not known. This prompted us to examine in more detail the stem growth kinetics of the three *Arabidopsis* accessions. 

### 2.5. Responses to High Salinity and Kinetics in the Stem Growth

**Figure 6:**
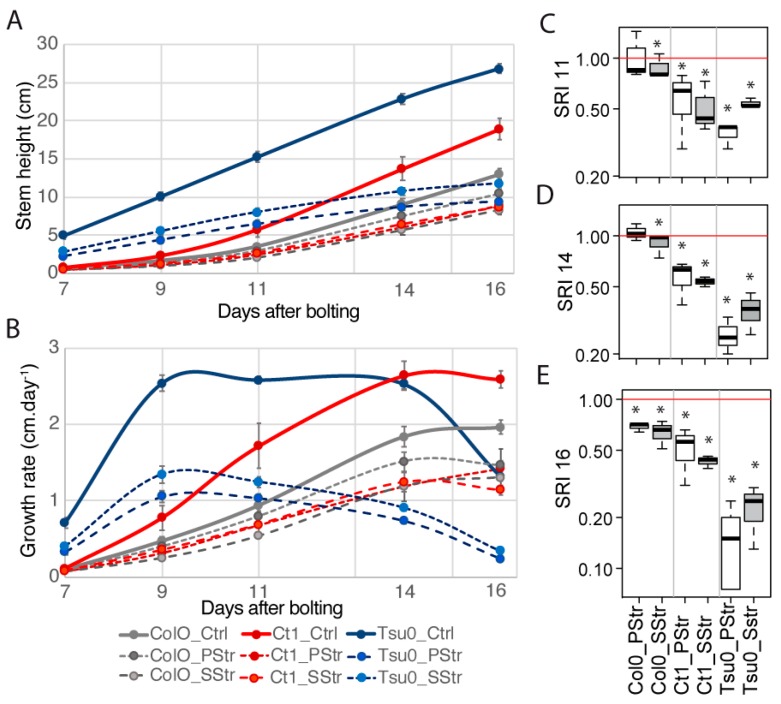
Stem growth in response to high salinity in *Arabidopsis*. Growth of the stem for the Ctrl, PStr, and SStr plants. The height of the stem was recorded at 7, 9, 11, 14, and 16 DAB. (**A**) The height of the stem and (**B**) the stem daily growth rate. The curves represent the mean ± SE (*n* = 9–18) for each time point. (**C**–**E**) The box and whisker plots represent the distribution of the biological replicates (see Figure 2 for details). (**C**–**E**) Salt response index (SRI) of the main stem at 11 (**C**), 14 (**D**), and 16 DAB (**E**). Stars denote significant differences of the treatments compared to the control plants (* *p* < 0.05, *n* ≥ 9). In white—PStr plants. In grey—SStr plant.

The stem height and its growth rate were examined from 7 to 16 DAB (Figure 6A,B). The growth rate of the Ctrl plants was smaller in Col0 compared with Ct1 and Tsu0 (Figure 6B). The growth rate of Col0 was not impacted much by the salt stress. In the Ct1 plants, in which the overall growth rate was higher than in the Col0 plants, the rate was slightly reduced under salt treatment from 7 to 16 DAB. Both in the Ctrl and salt-treated Col0 and Ct1 plants, this rate reached a plateau after 14 DAB. In Tsu0, the slope of the growth rate curve was different both in the Ctrl and salt-treated plants from the one observed in the two other accessions. It was characterized by higher rates at early time points, and then a plateau from 9 to 14 DAB, followed by a decline, both in the Ctrl and salt-treated Tsu0 plants. 

We then calculated the salt response index (SRI) at 11, 14, and 16 DAB (Figure 6C–E). Until 14 DAB, high salinity had little effect on Col0, whose SRI was close to 1 for the salt-treated plants. For Ct1 and Tsu0, whose stems grew faster during the two first weeks, we observed a smaller SRI at 11, 14, and 16 DAB. The SRI values in the salt-treated plants decreased with time (Figure 6C–E), with the smaller indexes being observed in the salt-treated Tsu0 plants.

### 2.6. Natural Variation in the Anatomy of the Stem in Normal and High Salinity Conditions 

**Figure 7:**
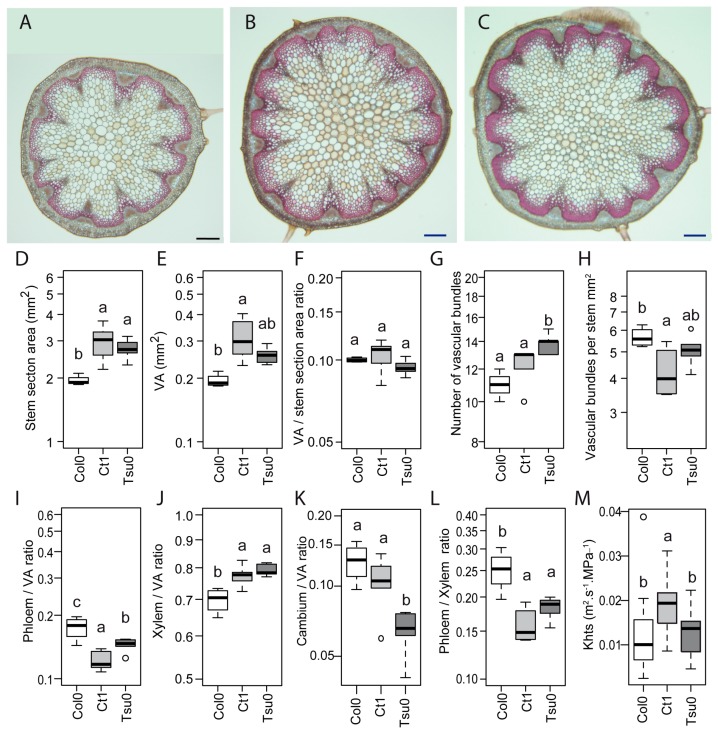
Natural variation of the anatomy in the stem of *Arabidopsis* plants at 17 DAB. (**A**–**C**) Representative stem sections of the Col0 (**A**), Ct1 (**B**) and Tsu0 (**C**) plants, with counterstaining with Safranin O and Alcian Blue. Bars = 200 µm. (**D**–**M**) The box and whisker plots represent the distribution of the biological replicates (see Figure 2 for details), for the stem section area (**D**), vascular area per stem section (VA) (**E**), vascular area/stem section area ratio (**F**), number of vascular bundles per section (**G**), number of vascular bundles per stem section area (**H**), phloem area/vascular area ratio (**I**), xylem area/vascular area ratio (**J**), cambium area/vascular area ratio (**K**), phloem area/xylem area ratio (**L**), and theoretical specific hydraulic conductivity (Khts) (**M**). Different letters indicate significant differences among accessions determined using a one-way analysis of variance (ANOVA) combined with Tukey’s comparison post-test (*p* < 0.05, *n* = 4–6, except for Khts, with *n* > 45).

To identify the additional clues explaining the different responses observed in the susceptible and tolerant accessions to a high salinity, we also analyzed the anatomy of the stem, looking at the stem sections sampled at the base of the stem. In the Ctrl plants, the area of the stem section was smaller in Col0 compared with Ct1 and Tsu0 (Figure 7A–C), with a smaller area of the vascular tissues (VA) (Figure 7D,E). However, the vascular area-to-stem area ratio remained similar in all three accessions (Figure 7F). Some variability was observed in the number of vascular bundles and in the number of vascular bundles per stem section area (Figure 7G,H). Finally, we observed a variability in the proportions of the phloem, xylem, and cambium areas in the vascular tissues (Figure 7I–L). The lumen area of the largest xylem vessels of each vascular bundle per stem section were measured in order to determine their diameter (D). Significantly higher D values were observed in the Ct1 plants compared with the Col0 and Tsu0 plants (18 µm ± 5, in Col0 (±SD, *n* = 177), 23.9 µm ± 4 in Ct1 (±SD, *n* = 296), and 19.2 µm ± 4 inTsu0 (±SD, *n* = 320)). As a result, the theoretical hydraulic specific conductivity of the stem was higher in the Ctrl Ct1 plants compared with the Col0 and Tsu0 plants, with the average values ranging from 0.01 to 0.02 m^2^. s^−1^. MPa^−1^ (Figure 7M).

**Figure 8:**
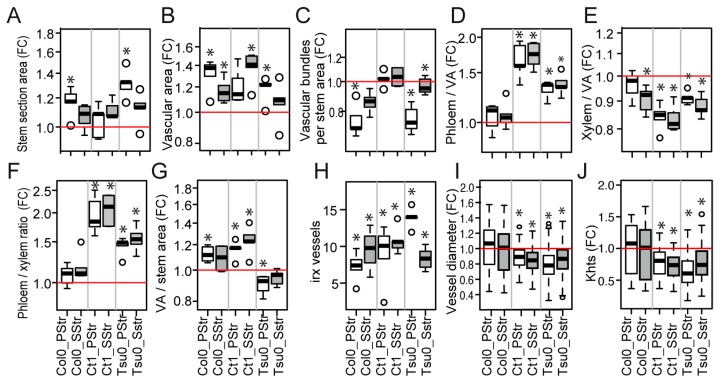
Natural variation in the *Arabidopsis* stem anatomy in response to a high salinity at 17 DAB. (**A**–**I**) The box and whisker plots represent the distribution of the biological replicates (see Figure 2 for details), within the (**A**) fold changes in the stem section areas, (**B**) the fold changes in the total vascular areas per stem section (VA), (**C**) the fold changes in the number of vascular poles per stem section surface unit, (**D**) the fold changes in the total phloem area/VA ratio per stem section, (**E**) the fold changes in the total xylem area/VA ratio per stem section, (**G**) the fold changes in the phloem area-to-xylem-area ratio, (**G**) the fold changes in the VA per surface unit of the stem section, (**H**) the number of irregular xylem (irx) vessels per vascular bundle, (**I**) the fold changes in the xylem vessel lumen area (**J**), and the fold changes in theoretical hydraulic specific conductivity. In grey—SStr plants. In white—PStr plants. FC—fold changes. Stars denote significant differences of salt treatment compared to the control condition (* *p* < 0.05, *n* = 4–6, except for the irx vessels and Khts values, with n > 36).

The same anatomic traits were analyzed in the salt-treated plants. Slight variations were observed in the stem section area (Figure 8A) and in the vascular area per stem section (Figure 8B). The number of vascular bundles per section area also showed slight variations both in the PStr Col0 plants and the PStr and SStr Tsu0 plants, compared with their respective Ctrl plants (Figure 8C). But the most significant effects were the higher proportions of phloem and the lower proportions of xylem in the vascular tissues in the salt-treated Ct1 and Tsu0 plants compared with the control plants (Figure 8D–F). Variations in the proportion of the vascular areas per stem section were also observed (Figure 8G). The diameters of the largest vessels and theoretical hydraulic specific conductivity that were calculated from the lumen area of the largest vessels were smaller in the salt-treated plants compared with the controls in the two tolerant accessions, Ct1 and Tsu0, (Figure 8I,J), while in Col0 plants, the high salinity had no effect on the D and Khts, compared with the Ctrl plants.

**Figure 9:**
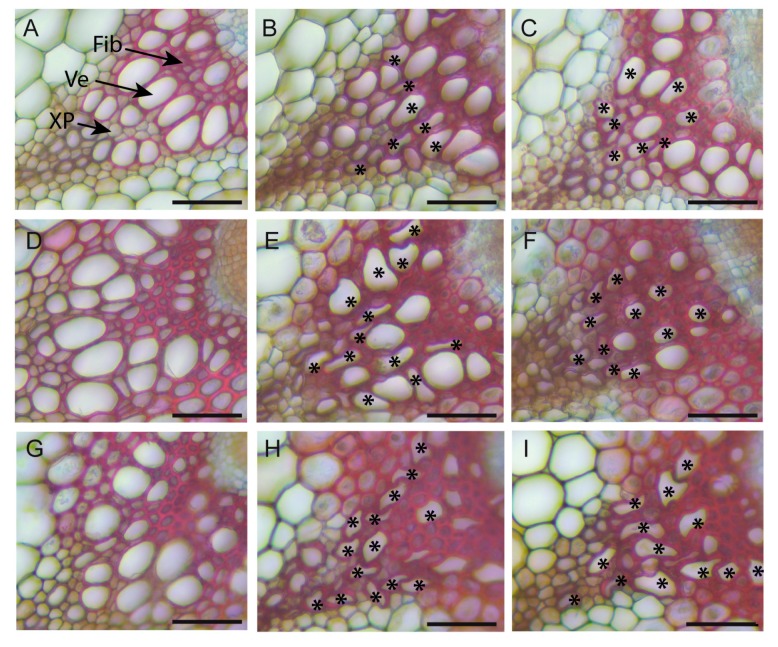
Observation of irregular xylem vessels under high salinity in the stem of *Arabidopsis*. Details of the xylem cells in the vascular bundles of Ctrl, PStr, and SStr plants at 17 DAB, observed in the stem section after staining with Safranin O and Alcian Blue. (**A**,**D**,**G**) Ctrl Plants, (**B**,**E**,**H**) PStr plants, (**C**,**F**,**I**), and SStr plants for Col0 (**A**–**C**), Ct1 (**D**–**F**), and Tsu0 (**G**–**I**). Stars (*) indicate irx vessels. Ve—vessel. Fib—xylem fiber. XP—xylem parenchyma cell. Bars = 50 µm.

A number of irregular xylem vessels were observed in the salt-treated plants, although they were not observed in the Ctrl plants (Figure 9), with less marked cell deformations in the salt-treated Col0 compared with Ct1 and Tsu0. These irregular xylem cells correspond to a phenotype known as “irregular xylem” (irx), found in a range of mutants deficient in secondary cell wall formation [36]. Under a high salinity, irx cells were observed both in the tolerant and susceptible *Arabidopsis* accessions, questioning the role of this trait in its adaptation to salt stress. No irx cells were observed in *Thellungiella* under our high salinity conditions.

### 2.7. Contrasted Responses of the Traits Related to Sugar Homeostasis and Anatomy in the Stem 

**Figure 10:**
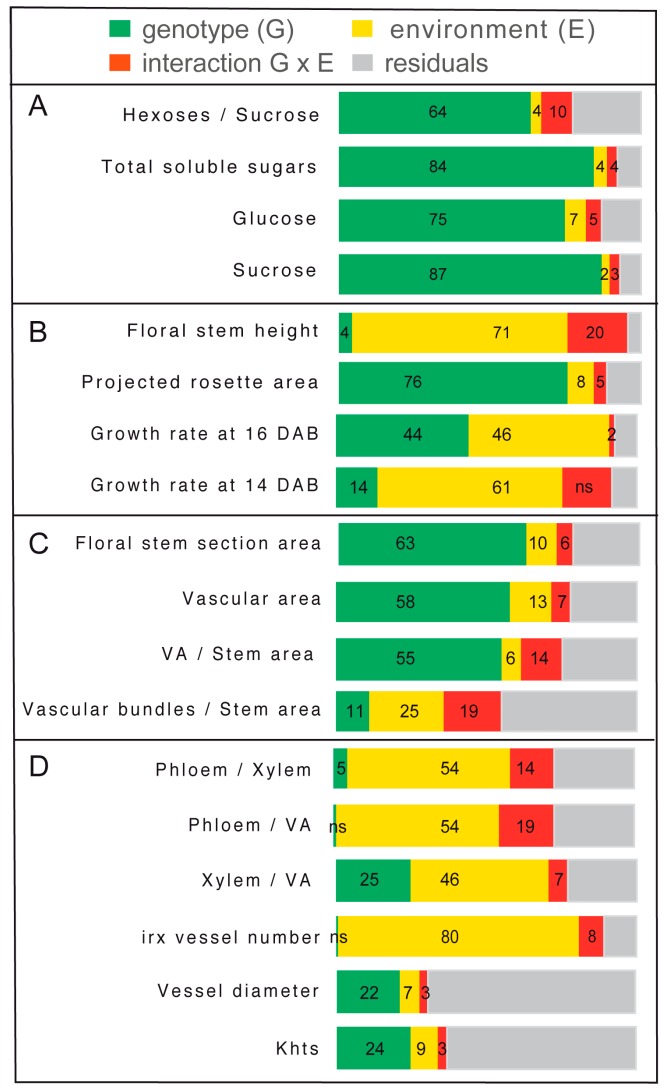
Analysis of variance of the traits measured during the reproductive stage, showing the effects associated with the genotype (G), the environment (E), and the interaction of genotype x environment (GxE) within the overall variations. VA—total vascular area per stem section. irx—number of irregular xylem cells per xylem section. Khts—theoretical hydraulic specific conductivity. For more details on the data, see the legends of Figure 3, Figure 4 and Figure 6. For each trait, the bars represent the sum of the squares associated with each factor, shown as proportions. The percentages of factors showing significant effects (*p* < 0.05) are indicated directly in the graph (ns for not significant effects). (**A**) Traits relative to the stem sugar content. (**B**) Growth traits of the rosette and stem. (**C**) Stem anatomic traits. (**D**) Traits relative to the stem vascular tissues.

The analyses of the variance of the different traits measured in response to the salt treatment confirmed both a significant genotypic effect and an effect of the salt environment (Figure 10). We observed predominant genotype effects on the stem sugar content, rosette growth, and stem section areas (Figure 10A–C), while the environment effects were predominate on the stem height and growth rate (Figure 10B,D). The largest genotypic effects were associated with the sucrose content in the stem (87% of variance), and the largest environmental effects were associated with the stem height (71% of variance) and occurrence of irx vessels (80% of variance). Several traits showed a significant effect of the interaction genotype x environment, including the stem height (20% of variance), the proportion of phloem within the vascular tissues (19%), and the number of vascular bundles per stem section (19%). For the two traits associated with water transport (i.e., diameter of the largest vessels and theoretical hydraulic specific conductivity, i.e. Khts), we observed an effect of the genotype, but minor effects of the environment or of the interaction genotype x environment. 

## 3. Discussion

In this study, we focused on the effects of high salinity during the reproductive stage, with an emphasis on the response in the floral stem. This approach aimed at examining the effects of the salt treatment on both the sugar content in the stem and the anatomy of the vascular tissues within the stem. The *Arabidopsis* floral stem plays an important role in carbon allocation. In addition, it is an interesting model to follow the effect of the stress on the development of the vascular tissues. By examining the natural variation of these responses between accessions, we aimed to identify the traits that developed in the accessions adaptated to high salinity.

In the three *Arabidopsis* accessions chosen for this study, which included both susceptible and tolerant accessions, a natural variability was observed in the sugar content, hexoses-to-sucrose ratio (Figure 3), anatomy of the stem and vasculature (Figure 7), and growth kinetics of the control plants (Figure 6). Despite this broad natural variation, *Arabidopsis*, like other herbaceous species, is generally described as a ruderal. Ruderal species reproduce quickly and use their nutritional resources for the optimal production and dispersal of seeds. By contrast, *Thellungiella* spp, which have a slow growth rate, are typical of “stress tolerator” species, according to the Grime’s classification [37]. When applying the salt treatment during the reproductive stage of *Thelungiella,* the stem growth was not impacted much by the 150 mM NaCl treatment (Figure 2). In contrast, we observed significant effects of salinity on the stem growth of the *Arabidopsis* accessions (Figure 4 and Figure 6), with a larger stem growth reduction in the two tolerant accessions, Tsu0 an then Ct1, while they had stronger growth in the control conditions. There were also major differences in the stem growth kinetics of the three accessions, with a variability in their growth rates (Figure 6 and Figure 10). Because of the strong effect of the interaction genotype per environment in explaining the variability of the stem height, this quantitative trait appears to be adaptive in *Arabidopsis* (Figure 10). 

Our findings also indirectly indicate that the extent of the stem growth reduction is not determined by the rosette biomass at the beginning of the salt treatment. Nor was it associated with the extent of the rosette growth reduction during the treatment (Figure 8). Thus, the strategy of the *Arabidopsis* tolerant accessions to cope with high salinity may be that the fitness, for example seed production, is improved by reducing the stem growth, while accumulating more sugars (Figure 5), a hypothesis that could be explored using the same experimental design set up for this work. Alternatively, another strategy to maintain seed production may primarily be to promote high vigor during the vegetative phase.

The vascular tissues in the stem are the main pathways for water and nutrient supply to the fruits. So far, few studies have explored the natural variation in the anatomy of the vascular tissues, and even less attention has been focused on any natural variation in response to biotic or abiotic stresses. Earlier studies only explored the xylem-to-phloem ratio in the hypocotyl under standard conditions [38], and the root anatomy in relation to the hydraulic parameters, with or without salt stress [15]. Here, under a high salinity, we observed a natural variation in the total areas of the stem section and vascular tissues (Figure 8), however most of the variability was explained by a genotypic effect (Figure 10), and our data provided no evidence that these changes were adaptive. In contrast, we found diversity among the accessions in the phloem-to-xylem ratio (Figure 8). This quantitative trait showed a strong effect of the genotype x environment interaction (Figure 10). The Ct1 and Tsu0 tolerant accessions displayed a lower proportion of xylem tissue per vascular bundle, and a higher proportion of phloem tissue per vascular bundle, in stress vs. control conditions (Figure 10). A major effect of the interaction (nearly 20% of the variance) was also found for the proportion of phloem within the vascular tissues (phloem-to-VA ratio) (Figure 10). Such changes potentially affect the nutrient long-distance transport capacity by maintaining carbon allocation via the phloem, while counteracting the water deficit and osmotic stress due to the salt.

Previous reports have shown a reduction of the xylem water transport in glycophytic plants under a high salinity. This was related to the changes in the root conductivity in cucumber, rice, and *Arabidopsis* [15,39,40]; the lumen areas of the stem vessels in poplar [41,42]; and the hydraulic conductivity in Eucalyptus [43]. In the *Arabidopsis* stem, we observed smaller lumen areas (A) and diameters (D) of the main vessels in the xylem (Figure 8), associated with smaller predicted values of the theoretical hydraulic conductivity (Khts) of the tolerant accessions Ct1 and Tsu0 under a high salinity (Figure 8). Interestingly, the lumen areas and diameters of the largest vessels and the predicted Khts values for *Thellungiella salsuginea*, both in the control conditions and in response to salt treatment (Figure 2), were much smaller than the ones observed in the *Arabidopsis* accessions, supporting the hypothesis that a reduction of Khts in the stem is an adaptive process. Differences in the stem anatomy and its predicted hydraulic profile could thus contribute to the variability in salt tolerance and provide clues about the adaptive processes. Another underlying issue is the relationship between the various traits analyzed in the stem and the traits associated with root anatomy and hydraulics, which should be further explored.

We also observed deformations of the xylem vessels in the stem of the three *Arabidopsis* accessions under high salinity that correspond to an irregular xylem (irx) phenotype, while none were observed in *Thellungiella salsuginea* (Figure 2 and Figure 9). Similar collapsed and deformed xylem vessels have been described in *irx Arabidopsis* mutants, and are indicative of defects in the secondary cell wall formation [36]. We do not know whether the irx phenotype observed in response to salt treatment is associated with defects in the xylan, lignin, or cellulose deposits in the vessel’s cell walls, as shown in many *irx* mutants [36]. Nevertheless, a recent transcriptomic study of the salt response of the inflorescence of the Col0 accession showed a transcriptional reprogramming of the genes involved in lignin biosynthesis, leading to a lower lignin content in the stem in response to the salt treatment [24]. The secondary cell wall defects observed under a high salinity in the stem and their contribution to salt tolerance should therefore be further investigated.

In *Arabidopsis*, the differences in the stem growth rate and vasculature anatomy under salt stress raise the question of whether sugar homeostasis in the stem is regulated. We indeed observed a natural variation in the stem sugar content and hexoses-to-sucrose ratios, both in the control conditions and in response to salt stress (Figure 3 and Figure 5). This latter quantitative trait showed significant genotype x environment interaction effects (Figure 10), suggesting that it may contribute to adaptive processes. Variations in the sugar content of the rosette are well known [33,34]. However few studies analyzed the sugar status in the floral stem, despite its major contribution to the plant’s lifetime carbon gain, and its importance for overall plant fitness [27,29]. Further studies are now required on the association between these traits and the regulation of the expression of genes involved in sugar transport and homeostasis. 

In conclusion, by describing a significant genetic variation of several traits related to the stem anatomy, this study provides a basis for the future quantitative genetic analyses of the physiological and anatomical traits of the stem involved in tolerance to osmotic stresses.

## 4. Materials and Methods 

### 4.1. Plant Material and Growth Conditions 

*Arabidopsis thaliana* accessions Columbia-0 (Col0), Catania-1 (Ct1), and Tsushima-0 (Tsu0), and the halophyte *Thellungiella salsuginea* were obtained from the Versailles Stock Center. The seeds were surface sterilized then sown in soil and grown in a growth chamber with a long-day photoperiod (150 µEm^−2^ s^−1^, 65% humidity, 16 h light at 21 °C/8 h dark at 17 °C). To synchronize the floral bud appearance, Col0, Ct1, and *Thellungiella salsuginea* were sown 7, 9, and 10 days, respectively, after the sowing of Tsu0 (Figure 1). The plants were watered three times per week by immersion in standard nutrient solution for four hours (10 mM nitrate, 2.75 mM potassium, 0.5 mM calcium, 0.7 mM chloride, and 0.25 mM phosphate) [44].

### 4.2. Salt Treatment and Pretreatment (Acclimation)

For the salt treatment, the plants were watered three days before the onset of floral bud by immersion in a nutrient solution containing 150 mM NaCl, for 6 h, in order to obtain the full imbibition of soil. The plants were then watered three times per week for 19 days with the salt nutrient solution. For acclimation, the plants were submitted to progressive pre-treatment of 25 mM for three days, and 50 mM NaCl for two days, before the beginning of the 150 mM salt stress (Figure 1). 

### 4.3. Growth and Physiological Parameters

The plant growth was recorded daily after the beginning of the acclimation and salt stress. The projected rosette area was measured daily from the pictures taken from day 1 to day 13 after the beginning of acclimation, and were analyzed using ImageJ Software (https://imagej.nih.gov/ij/), with five to six replicates per genotype and per condition. The height of the main floral stem was measured every two or three days from 5 to 16 DAB, with at least nine replicates per genotype and per condition. The daily growth rate of the floral stem (plotted at the end of the interval on Figure 2 and Figure 6) was determined as the increase in height between two time points, divided by the number of the days of the interval. The salt response index (SRI) was calculated by dividing the stem growth rate of the PStr or SStr plants, by the mean stem growth rate of the Ctrl plants (equivalent of the osmotic tolerance index described by Rajendran and colleagues [32]). The destructive measures of the fresh weight (FW) and dry weight (DW) of the rosette and floral stem were made at 16 DAB, with four replicates per genotype and per condition. The relative water content was calculated as the ratio (FW − DW)/FW. 

### 4.4. Carbohydrate Content

The fully expanded rosette leaves and floral stem, without lateral stems, siliques, flowers, and cauline leaves, were harvested at 17 DAB, eight hours after the beginning of the light period, and were frozen in liquid nitrogen before grinding. The soluble sugars were extracted from 50 mg of frozen powder, by two successive additions of 80% ethanol, for 2 h in ice. The supernatants after centrifugation were separated from the residual solid material, evaporated with a speed-vac, and re-suspended in water [45]. The sucrose and hexose levels were determined using an enzymatic sucrose/D-glucose/D-fructose kit (R-Biopharm, Darmstadt, Germany). For each genotype, five to six biological replicates were analyzed per condition.

### 4.5. Anatomy of Stem Sections

The floral stems harvested at 17 DAB for the analysis for the carbohydrate content were used for anatomic studies. The first cm of the basal part of the main floral stem was collected and embedded in 8% low-melting agarose. For each genotype, four to six biological replicates were analyzed per condition. The transverse sections (50 µm thick) were cut on a Vibratome MM France, and were stored in 70% ethanol at 4 °C until use. For the imaging, the sections were colored for 20 s with Safranin O–Alcian Blue staining [46]. The observations were done by Zeiss Stereo Microscopy using a color camera and transmitted light. Using ImageJ Software, the following parameters were measured: the area of the stem cross section, number of vascular bundles, and total area of the vascular bundles. For each vascular bundle, the total areas of the phloem, xylem, and cambium were measured. For each xylem pole, the number of irregular xylem cells was determined with the cells counter function of the “Analyze” plugin of ImageJ (http://imagej.nih.gov/ij/), with at least four to five biological replicates per genotype and condition, one section per biological replicate, and the irx cells determined for at least nine poles per section.

### 4.6. Stem Theoretical Hydraulic Specific Conductivity

The theoretical hydraulic specific conductivity (Khts) was predicted from the Hagen–Poiseuille equation, using the following dimensions of the largest vessels [47]: for each individual vascular bundle in each stem section, the lumen area (A) of three to four xylem vessels was measured. The circle diameter (D) of each vessel was deduced from A, as D = (2√(A/π)) [48]. Then, the Khts was determined as Σ(πD^4^/128η)/ΣA, where η is the water viscosity coefficient at 20 °C (1.0016 mPa/s) and D is the circle diameter [47]. For each genotype, four to six biological replicates were analyzed per condition, and for each section, at least nine poles per section were analyzed, except for *Thellungiella*, for which three to four poles were analyzed. 

### 4.7. Statistical Analyses

The statistical analyses were performed using a two-way ANOVA combined with a Tukey’s comparison post-test, using R statistical software, version 3.3.2 (http://www.r-project.org). A *p-*value of <0.05 was considered significant.
